# Red light-induced structure changes in phytochrome A from *Pisum sativum*

**DOI:** 10.1038/s41598-021-82544-2

**Published:** 2021-02-02

**Authors:** Mao Oide, Masayoshi Nakasako

**Affiliations:** 1 Department of Physics, Faculty of Science and Technology, Keio University, 3-14-1 Hiyoshi, Kohoku-ku, Yokohama, Kanagawa 223-8522 Japan; 2 RIKEN, SPring-8 Center, 1-1-1 Kouto, Sayo-cho, Sayo-gun, Hyogo 679-5148 Japan

**Keywords:** SAXS, Light responses

## Abstract

Phytochrome A (phyA) is a photoreceptor protein of plants that regulates the red/far-red light photomorphogenic responses of plants essential for growth and development. PhyA, composed of approximately 1100 amino acid residues, folds into photosensory and output signaling modules. The photosensory module covalently binds phytochromobilin as a chromophore for photoreversible interconversion between inactive red light-absorbing (Pr) and active far-red light-absorbing (Pfr) forms to act as a light-driven phosphorylation enzyme. To understand the molecular mechanism in the initial process of photomorphogenic response, we studied the molecular structures of large phyA (LphyA) from *Pisum sativum*, which lacks the 52 residues in the N-terminal, by small-angle X-ray scattering combined with multivariate analyses applied to molecular models predicted from the scattering profiles. According to our analyses, Pr was in a dimer and had a four-leaf shape, and the subunit was approximated as a bent rod of 175 × 50 Å. The scattering profile of Pfr was calculated from that recorded for a mixture of Pr and Pfr under red-light irradiation by using their population determined from the absorption spectrum. The Pfr dimer exhibited a butterfly shape composed of subunits with a straight rod of 175 × 50 Å. The shape differences between Pr and Pfr indicated conformational changes in the Pr/Pfr interconversion which would be essential to the interaction with protein molecules involved in transcriptional control.

## Introduction

Plants have red/far-red light receptor protein, phytochrome, for sensing environmental conditions to regulate their growth and development^1^. After the discovery of phytochrome A (phyA) as a photoreceptor for red/far-red reversible responses in seed germination^2^, five phytochromes (phyA, B, C, D, and E) were identified in most land plants, such as *Arabidopsis thaliana*^3^. In addition, with genomic developments, diverse phytochrome superfamilies of bacteriophytochromes (Bphs) have been identified in bacteria and alga^4^. Red light-activated plant phytochromes cause a broad range of photomorphogenic responses, such as seed germination, de-etiolation, and floral induction^5–7^, by inducing genome-wide changes in alternative promoter selection to modulate protein localization^8^.

**Figure 1:**
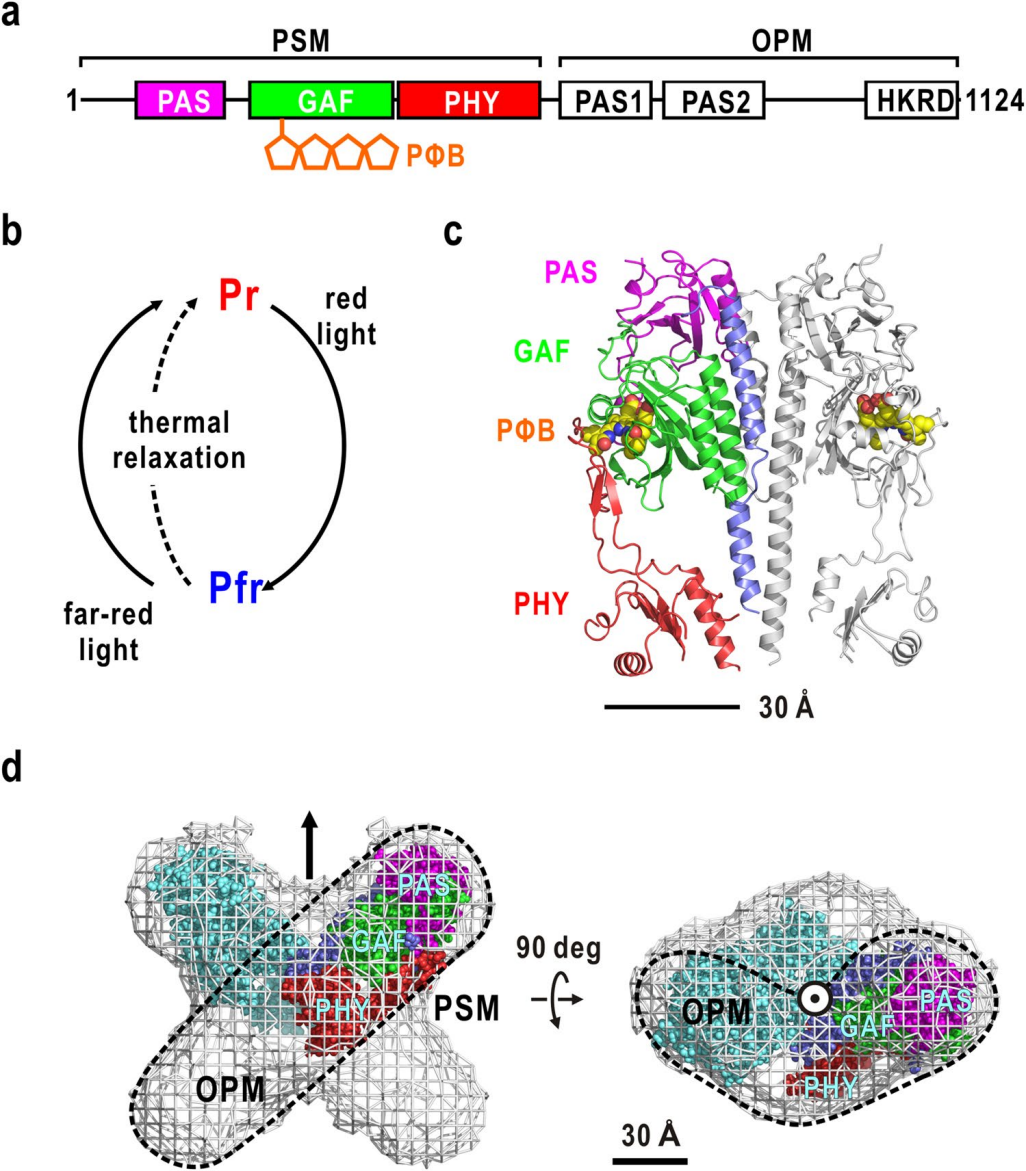
(**a**) Organization of functional domains and modules in *P. sativum* phyA. (**b**) Transformation of phytochrome. (**c**) Crystal structure of the PSM dimer of *A. thaliana* phyB (PDB accession code: 4OUR)^12^. Functional domains are colored according to the coloring scheme of domains in panel (**a**). (**d**) Molecular shape of *A. thaliana* phyB predicted from SAXS^37^. A volume encircled by a dashed line indicates a subunit. The subunit of the crystal structure of PSM of A. thaliana phyB is fitted. Panels (**c**, **d**) were drawn using the *PyMol* program^56^.

PhyA and phyB, major members of the plant phytochrome family, are soluble chromoproteins composed of approximately 1100 amino acid residues (molecular weight (*M*_w_) 125 kDa) and one linear tetrapyrrole chromophore, phytochromobilin (PΦB)^1^ (Fig. 1a). Phytochrome takes the inactive red light-absorbing (Pr) and active far-red light-absorbing (Pfr) forms^9^ (Fig. 1b). Pr and Pfr forms are photo-reversibly interconverted to each other, and Pfr reverts to Pr through thermal relaxation (dark reversion)^10^. Only Pfr has been believed to be biologically active, but Pr also contributes to biological responses, as revealed by the far-red light high irradiance responses of phyA^11^.

The structure–function relationships of phyA and phyB have been extensively studied. These molecules comprise the photosensory module (PSM) with a *M*_w_ of 70 kDa in the N-terminal, and the C-terminal output module (OPM) with a *M*_w_ of 55 kDa (Fig. 1b)^1^. From the N- to C-terminal of PSM^12^, the N-terminal extension (NTE), Period/Arnt/Sim domain (PAS)^13^, cGMP phosphodiesterase/adenylyl cyclase/FhlA domain (GAF)^14^, and phytochrome-specific domain (PHY)^15^ are tandemly arranged (Fig. 1c). The NTE interacts with part of the GAF and is correlated with the stability of light-activated phytochromes^16^. GAF, which has a primary structure similar to hundreds of signaling and sensory proteins, covalently binds PΦB by its lyase activity for light-sensing and reversible Pr/Pfr photo-transformation^17^. PHY is essential to keep the absorption spectra intact through interactions with GAF^12^, and also plays an important role for stabilizing the Pfr of phyB^18^. Photoconversion involves the isomerization within PΦB^9^, but the molecular mechanism that activates phytochrome is currently unclear.

OPM is shared by two PAS on the N-terminal side and a histidine kinase-related domain (HKRD) on the C-terminal side, and plays major roles in dimerization, nuclear import, and localization^1^. In phyB, OPM acts as an attenuator of phytochrome activity, since PSM dimer can trigger full phyB responses with much higher photosensitivity than the full-length phyB in the nucleus^19^. Two PAS contain amino acid residues essential for signal transduction from PSM to OPM^20^. HKRD, a paralog of histidine kinase, displays Ser/Thr kinase specificity in contrast to the histidine kinase function of Bphs^21^, and overlaps with the nuclear localization signal sequence^22^. Pfr interacts with proteins necessary for nuclear localization and signaling partners, such as the phytochrome-interacting factor (PIF) family^23^ and ubiquitin ligase complex^24^. In contrast to PSM, the structures of domains in OPM are currently unknown, and the prediction of their tertiary structures remains a challenge due to there being little structural information available regarding proteins with primary sequences homologous to OPM domains.

With respect to higher plants, the atomic structure is only known for the PSM of *A. thaliana* phyB^12^. The structures of full-length and PSMs of Bphs have been insightfully studied by X-ray crystallography^25–35^. The structures of PSM are similar among Bphs with respect to the arrangements of PAS, GAF, and PHY. However, the PSM dimers found in crystals are in different association modes^30,31,35^, suggesting the influence of amino acid sequences and/or molecular contacts in crystals on the association of the PSM dimer. Red light-induced conformational changes around the chromophore and in the arrangement of functional domains have also been investigated for Bphs in crystals^27,29^. It is currently unclear how relevant the structural information of Bphs in crystals is to plant phytochromes in solution.

In contrast to crystallography, small-angle X-ray scattering (SAXS) allows the investigation of low-resolution structures of proteins in solution^36^. Regarding plant phytochromes, SAXS studies have been reported for the full-length phyB of *A. thaliana*^37^ and the large phyA (LphyA) of *Pisum sativum*^38,39^. LphyA lacks 52 amino acid residues of NTE (total 1072 residues) but displays the same photo-transformation pathway and physicochemical properties as full-length phyA^40^. The dimer of LphyA in Pr, with a partial specific volume comparable to standard soluble proteins, has a four-leaf shape, which could simulate the observed SAXS profile and explain the transmission electron microscopy (TEM) images^38^. After preparing a completely monodispersive solution of LphyA, we successfully recorded SAXS profiles from LphyA solutions in Pr and Pfr^39^. Then, by applying the ab initio algorithm to predict molecular shapes from SAXS profiles^41^, we obtained an averaged molecular shape of each form from only 10 independent calculations performed on a small computer system^39^.

Since the SAXS profile only contains the information of orientationally averaged structure, predicted molecular models generally suffer from an ambiguity in their shapes^42^. As a result, inappropriate models blur the averaged molecular shape^43^. To improve this, we proposed a protocol that incorporates multivariate analysis to appropriately extract the most probable shape from a large number of ab initio models, which are now easily obtainable through a high-performance computation system^43^. The protocol successfully predicted molecular shapes of *A. thaliana* phyB^37^ and *A. thaliana* phototropin 2^44^ from their SAXS profiles, and both shapes were consistent in terms of their TEM images.

As the number of models predicted in the previous study was too small to appropriately extract the most probable shape, here we performed a much larger number of ab initio calculations for the previously obtained SAXS profiles from Pr and Pfr of LphyA and utilized our protocol to extract the most probable shapes. As a result, we obtained a four-leaf shape of Pr, and a butterfly shape of Pfr, both of which topologically resembled the molecular shape of full-length phyB^37^. Additionally, the molecular shape of Pfr exhibited substantial differences with that of Pr, indicating conformational changes in the Pr/Pfr interconversion. Based on the new shapes, we discuss the arrangements of subunits and modules in Pr, and the rearrangement in Pfr for interactions with protein molecules involved in transcriptional control.

## Results

### SAXS of Pr and Pfr

**Figure 2:**
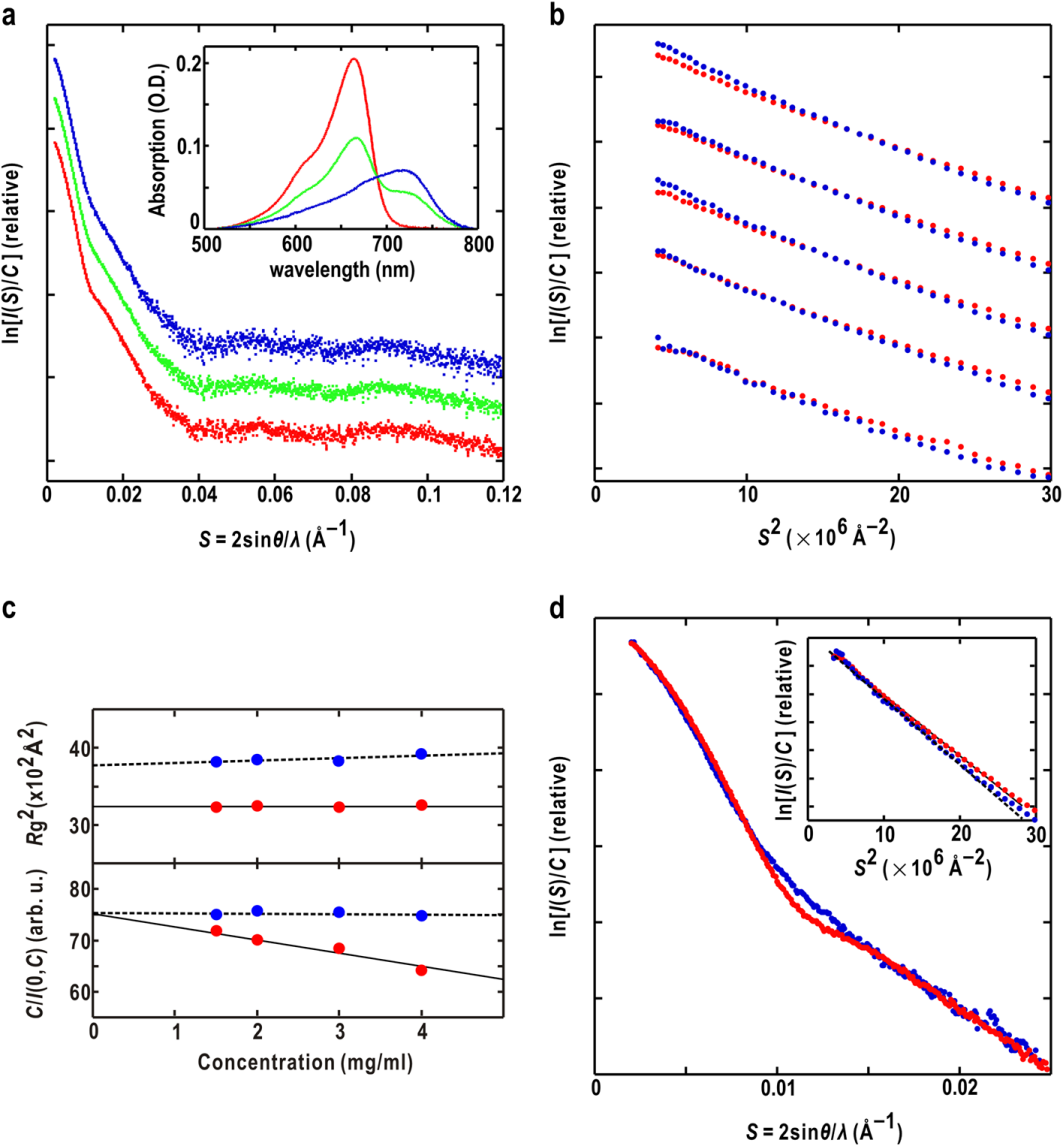
(**a**) SAXS profiles of Pr (red dots) under red light irradiation (green) and Pfr (blue) of LphyA. The Pfr profile was calculated based on the assumption that the population of Pr is 0.39 and the other of Pfr is 0.61, that was estimated from the absorption spectra of Pr (red line) and the steady state (green) in the inset. The Pfr absorption spectrum (blue in the inset) was calculated using the population ratio. (**b**) Guinier plots of Pr (red dots) and Pfr (blue) of LphyA solutions. From lower to upper, the concentrations of LphyA are 0.5, 1.5, 2.0, 3.0, and 4.0 mg/mL, respectively. The Guinier approximations were used for the region from 4 × 10^–6^ to 20 × 10^–6^ Å^2^. (**c**) Concentration dependencies of *R*g and the inverse of zero-angle scattering intensities of Pr (red symbols) and Pfr (blue). The variation of the parameters was approximated by linear regression lines. The signs for *A*_2_ of Eq. (4) and *B*_if_ of Eq. (5) are the same. (**d**) Scattering profiles of Pr (red dots) and Pfr (blue) near the dilution limit estimated from the profiles of the concentration series. The inset shows their Guinier plots.

In a previous study^39^, we obtained SAXS profiles up to a resolution of 7 Å from Pr and the steady state (Pr/Pfr mixture) at a concentration of 1–4 mg/mL (Fig. 2a). The SAXS profiles of Pr exhibited little aggregation as the Guinier plot approximated by single regression lines (Fig. 2b). Furthermore, the monodispersive property was confirmed by the linear concentration dependencies of the inverse of the zero-angle scattering intensities and the radii of gyration (Fig. 2c). SAXS profiles recorded 15 min after turning off red light were almost consistent with those of Pr in the dark, indicating the thermal relaxation from Pfr to Pr.

Since the absorption spectra of Pr and Pfr have overlaps, both forms are always present under red light irradiation (steady state) (inset in Fig. 2a). In the diluted solution, all LphyA dimers were separated sufficiently that the interference patterns between dimers within the spatial coherent length of the incident X-ray beam went into the beamstop. In the dark reversion, Pr-Pr homodimer, Pr-Pfr heterodimer, and Pfr-Pfr homodimers were present in solution. In contrast, in the Pr/Pfr photo-steady state under red light irradiation, the Pr-Pfr heterodimer immediately photo-converted either the Pr-Pr or Pfr-Pfr heterodimer, and the thermal reversion of Pfr-Pr heterodimer is reported to be much faster than that of Pfr-Pfr homodimer^45^. Therefore, we assumed that the population of Pr-Pfr dimers was small in the steady state, and the scattering profile of the Pr/Pfr photosteady state ($I_{{{\text{steady}}}} (S)$) was the sum of scattering intensities from Pr-Pr ($I_{\Pr \Pr } (S)$) and Pfr-Pfr ($I_{{{\text{P}} {\text{fr}}{\text{P}} {\text{fr}}}} (S)$) dimers as follows:

1 $$ I_{{{\text{steady}}}} (S) = \omega_{\Pr \Pr } I_{\Pr \Pr } (S) + \omega_{{{\text{P}} {\text{fr}}{\text{P}} {\text{fr}}}} I_{{{\text{P}} {\text{fr}}{\text{P}} {\text{fr}}}} (S) $$

2 $$ \omega_{\Pr \Pr } + \omega_{{{\text{P}} {\text{fr}}{\text{P}} {\text{fr}}}} = 1 $$

where $\omega_{\Pr \Pr }$ and $\omega_{{{\text{P}} {\text{fr}}{\text{P}} {\text{fr}}}}$ are the populations of the two types of dimers, and were 0.39 and 0.61, respectively, as determined from the absorption spectra of the steady state and Pr (Fig. 2a).

The SAXS profile of Pfr reconstructed using Eqs. (1) and (2) demonstrated a significant decrease in 0.004 < *S* < 0.008 Å^-1^ and an increase at around *S* = 0.011 Å^-1^ (Fig. 2a,d), suggesting red light induced conformational changes over the LphyA molecule. In addition to Pr, the monodispersive property of Pfr solution was confirmed by the Guinier plots (Fig. 2b), and the linear concentration dependencies of zero-angle scattering intensities and the radii of gyration (Fig. 2c). The radius of gyration of Pfr (62.6 ± 0.4 Å) was larger than that of Pr (57.1 ± 0.1 Å), suggesting the expansion of molecular size in Pfr (inset in Fig. 2d).

### Molecular shapes of Pr and Pfr

**Figure 3:**
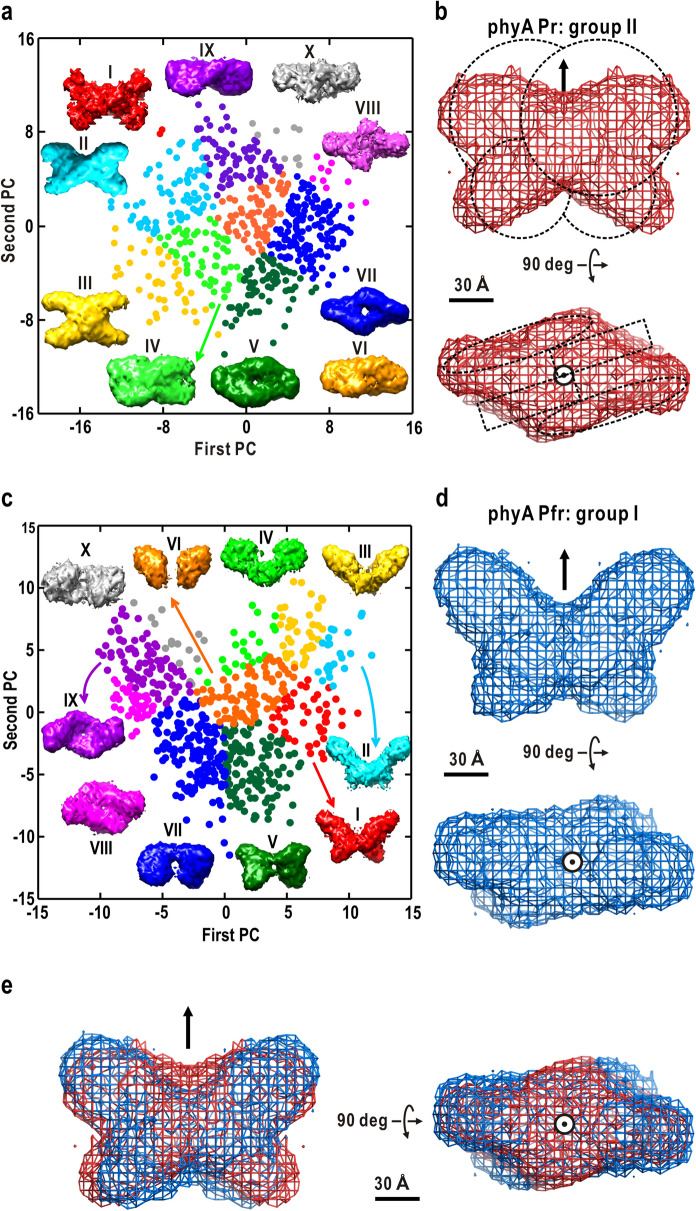
Selection of the most probable molecular shape of the Pr dimer (**a**, **b**) and Pfr dimer (**c**, **d**). In panels (**a**, **c**), 560 models from independent ab initio calculations are plotted on the plane spanned by the first and second eigenvectors from PCA. Through K-means clustering, the models were classified into 10 groups designated I-X. The number of the Pr models classified into groups I-X are 2, 75, 43, 59, 73, 77, 142, 10, 70, and 9, respectively. On the other hand, those of the Pfr models in groups I-X are 45, 23, 38, 23, 116, 84, 150, 41, 79, and 15, respectively. The molecular shape of each group was obtained by averaging the DR models after superimposition on a reference structure. The averaged shape for each group was illustrated using *UCSF Chimera*^57,58^. Panels (**b**, **d**) show averaged shapes for group II of Pr and group I of Pfr, respectively. The representing shapes are shown as mesh models with arrows and symbols representing the two-fold symmetry axes. In panel (**b**), the four-leaf shaped model (dashed lines) in the early stage of our SAXS study^38^ is superimposed. (**e**) Superimposition of averaged molecular shapes of Pr (red mesh) and Pfr (blue).

The SAXS profile of LphyA showed ambiguity scores^42^ of approximately 1.7, suggesting that molecular models predicted by the ab initio algorithm would be in variety, and that a set of only 10 ab initio calculations in the previous study^39^ was too small to identify probable molecular shapes. Here, we performed more than 500 independent ab initio calculations for each of the SAXS profiles of Pr and Pfr in Fig. 2a, and used the protocol, including multivariate analysis, to extract a set of the most probable models (Fig. 3). The optimum number of dummy residues (DRs) was 1000 for both Pr and Pfr and was different from the actual number of amino acid residues, probably due to flexible loops with low electron density contrast against the buffer solution^43^.

Figure 3a shows the distribution of Pr models on the plane spanned by the two principal components displaying the two largest eigenvalues in the principal component analysis (PCA). Among the 10 groups classified by K-means clustering, groups V-X with averaged shapes approximated as a pair of twisted tubes in anti-parallel association were major, and those with a cross shape (I-IV) were minor. According to the calculation by *EMBOSS Water*^46^, the sequence identity and homology between *A. thaliana* phyB and *P. sativum* phyA are 51.4% and 70.3%, respectively. These suggest that the molecular structure of LphyA is probably similar to that of phyB. In the previous SAXS study on phyB, the cross shape among the predicted models was selected for the molecular shape of phyB, and was validated by the electron microscopy images of negative-stained phyB (Fig. 1d)^37^. Therefore, we selected a cross shape (groups I-IV) as the most probable molecular shape of the Pr dimer. In addition, the presence and absence of cross-shaped models in ab initio calculation depended on the resolution of SAXS data. In fact, cross-shaped models are very minor in the ab initio calculations for the SAXS data of low resolution (up to a resolution of 0.038 Å^-1^), while they were second major in the present calculations used SAXS data up to 0.12 Å^-1^.

As reported in the previous study^37^, the averaged shape of group II displayed a cross shape with four equidimensional bulges from the main body (point-symmetric crossed shape). However, through careful inspection of the models in group II, we found that each model was anisotropic without the symmetry. Then, all models of the group II were carefully realigned by paying attention to their anisotropic shapes, and finally we obtained the shape representing group II by averaging over realigned models (Fig. 3b). The representing shape was characterized by two large and two small lobes radiating from the main body, and was superimposable onto the four-leaf shape proposed in our preliminary study^38^. Therefore, we also refer to the representing shape of group II, as a four-leaf shape. Scattering profiles calculated from the averaged shape simulated the experimental profile in S < 0.02 Å^-1^ (data not shown).

Regarding Pfr, 614 molecular models were roughly classified into three types; groups VIII and IX were composed of a pair of twisted tubes, groups V-VII and X were approximated as two large leaves, and groups I-IV with a butterfly shape (Fig. 3c). While the third was minor, we selected group I as the most probable shape due to the resemblance of the averaged shape of the group to that of Pr (Fig. 3d). The averaged molecular shape of group I simulated the experimental SAXS profile in S < 0.02 Å^-1^ (data not shown). The Pfr shape displayed substantial differences from that of Pr. The large lobe in Pr likely extended or shifted along the direction of the major axis, and the other two appeared to shift from their positions in Pr (Fig. 3e).

Although the new shapes had dimensions comparable to those of the previous Pr and Pfr models^39^, the shape was much clearer, probably due to excluding incorrect models blurring averaged shapes. The association mode of subunits, locations of functional modules, and conformational changes in the photoconversion will be discussed later.

### Normal mode analysis for the PSM of A. thaliana phyB

**Figure 4:**
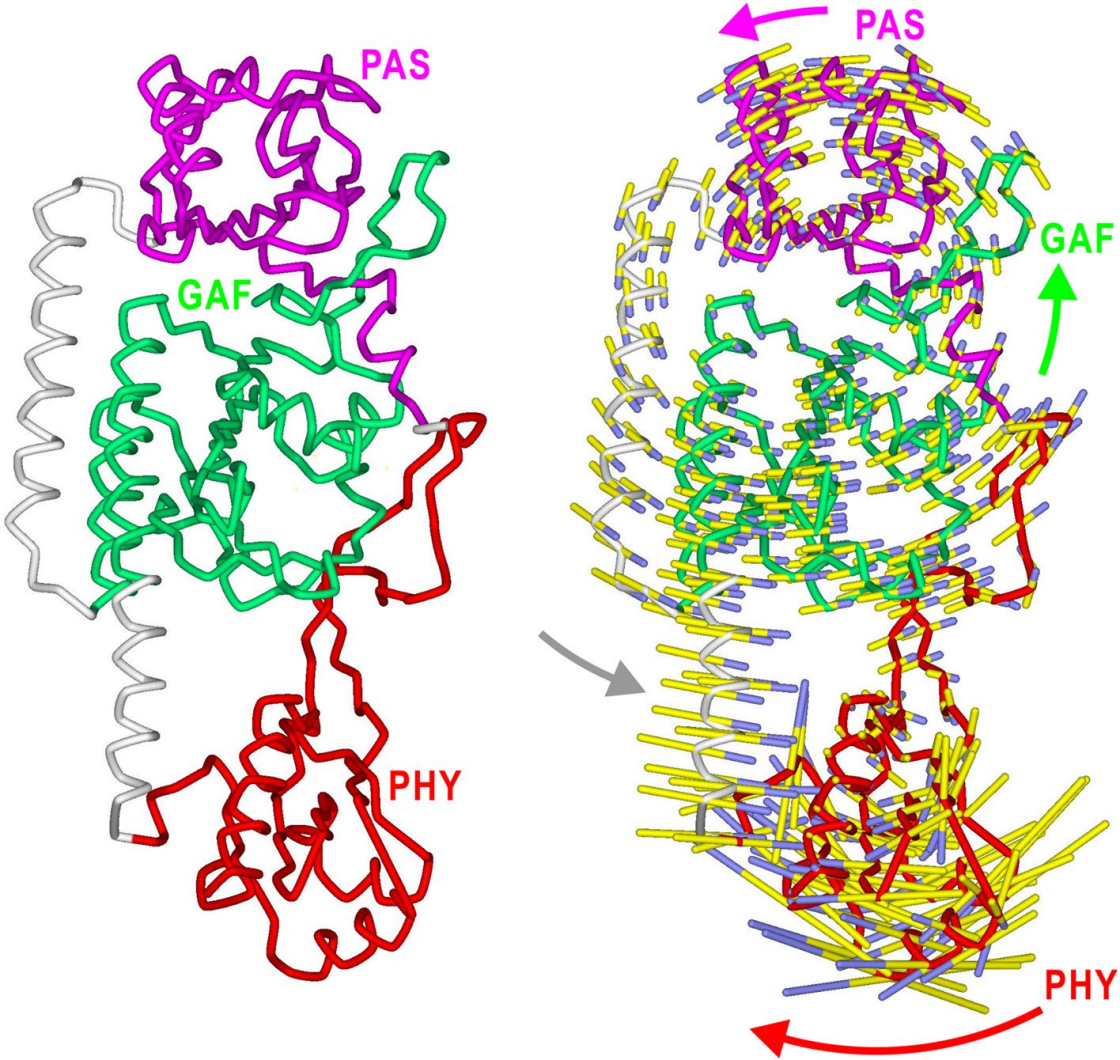
Right panel shows normal mode motions with the lowest energy in subunit A in the crystal structure (left panel) of PSM of *A. thaliana* phyB. The 3-times magnified amplitudes of the motions of Cα atoms are illustrated by colored sticks, which indicate the directions of the motions from the yellow to blue ends. The motions of domains are schematically indicated by arrows. The cutoff distance used in the normal mode calculation was 7 Å.

To understand the types of motions possible in PSM, we applied normal mode analysis^47,48^ to the elastic network model^48^ for the crystal structure of a PSM subunit (Fig. 4). Since molecular dynamics simulation is difficult to be applied to the crystal structure missing several parts, normal mode analysis is better to approximately illustrate possible motions. Owing to the similarity of amino acid sequences, the tertiary structure of PSM was expected to be similar between *P. sativum* phyA and *A. thaliana* phyB.

Figure 4 shows the first normal mode with the lowest energy, which displays the largest displacement. The normal mode motion is explained as the hinge-bending motion of PHY against the cluster of PAS, GAF, and the two long α-helices to bend the subunit. Therefore, the normal mode suggests that the PSM structure intrinsically possesses internal motions to drive PHY and probably the subsequent OPM.

## Discussion

In this study*,* we obtained molecular shapes of *P. sativum* LphyA in Pr and Pfr by applying multivariate analyses to a large number of ab initio models restored from the SAXS profiles. The differences between the molecular shapes of Pr and Pfr indicate that conformational changes in the Pr/Pfr interconversion accompany the rearrangement of subunits and modules. Here, we discuss the arrangement of subunits and functional modules in Pr and Pfr for better understanding of the molecular mechanism in the initial stage of photomorphogenesis in plants.

### Arrangement of subunits and functional modules

**Figure 5:**
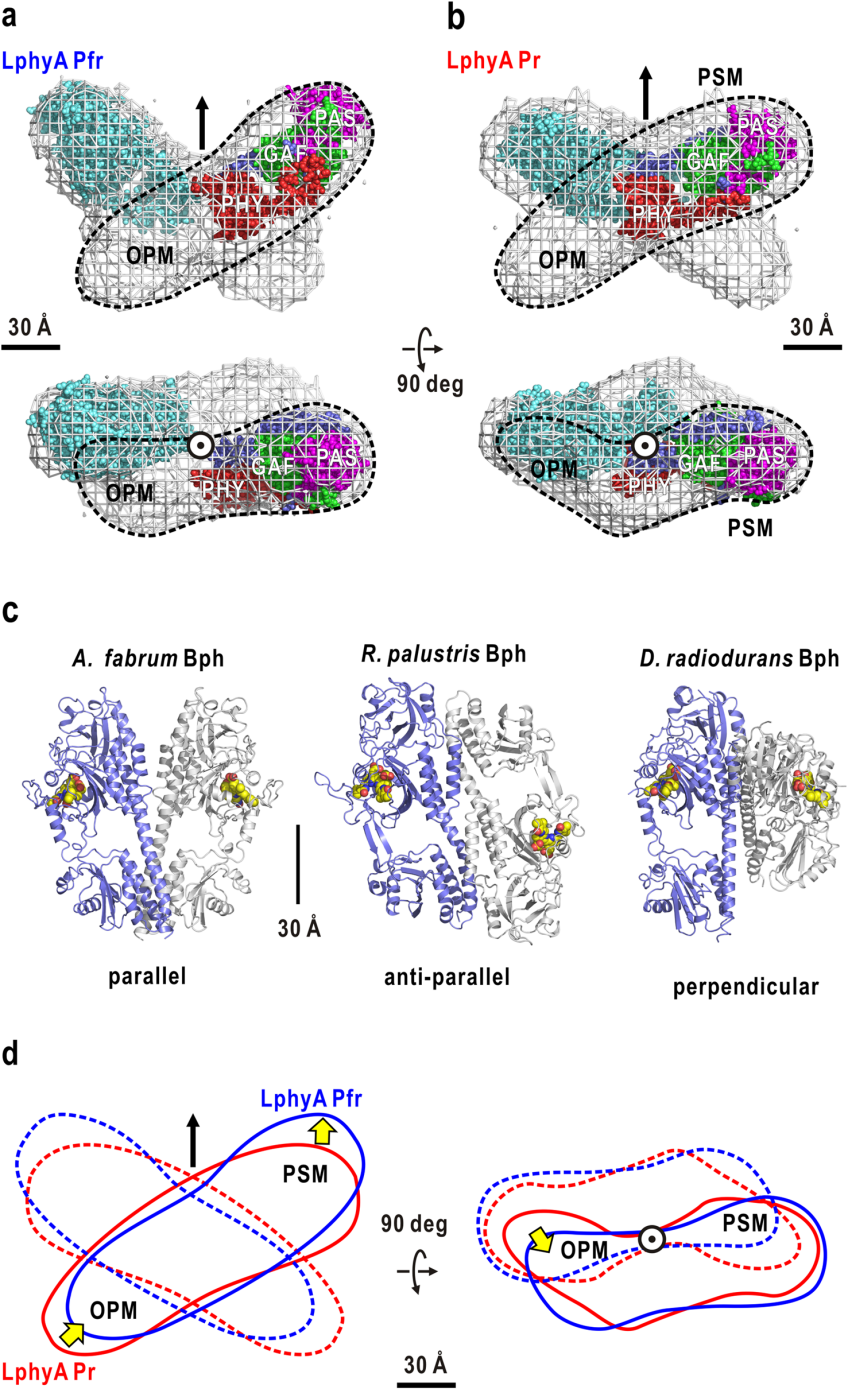
Most probable molecular models of the LphyA dimer in Pfr (**a**) and Pr (**b**). Averaged molecular shapes from Fig. 3 are illustrated as gray mesh. The dashed circles indicate one subunit. Subunit A in the crystal structure of PSM dimer from *A. thaliana* phyB (PDB accession code: 4OUR) is fitted to PSM volumes in each averaged model. The arrow in the upper panel and symbol in the lower indicate the two-fold symmetry axis in the dimer. Panels (**a**, **b**) were drawn using *PyMol*^56^. (**c**) Three types of association modes of PSM of Bph and phyB in their crystal structures. Two subunits are distinguished by the colors of the models. (**d**) Schematic illustration of structural changes in Pr/Pfr photoconversion. Yellow arrows indicate movements of the functional modules.

The Pfr shape displays the borders of the subunits clearer than the Pr shape, and can be illustrated as a crossed orientation of two subunits, each of which can be approximated as a straight rod of 175 Å long and 50 Å wide (Fig. 5a). The volume of the rod is sufficient to tandemly arrange the PSM and OPM along the direction of its major axis. For instance, the crystal structure of the phyB PSM subunit^12^ can be fitted to the upper part of the rod. The opposite arrangement of PSM and OPM from Fig. 5a is also possible. However, the locations of modules proposed in Fig. 5a may be consistent with the idea that OPM predominantly acts as a dimerization site^49^.

By referring to the subunit arrangement in Pfr, the border of the subunits in Pr could be defined as that in Pr (Fig. 5b). A subunit is approximated as a rod of 175 Å long and 50 Å wide. In contrast to the straight rod of Pfr, the rod of Pr has a bend at the middle. When arranging the crystal structure of the phyB PSM, the bend is likely located near the border of between PSM and OPM. The arrangements and the bent shapes of subunits in LphyA Pr are similar to those in the SAXS model of full-length *A. thaliana* phyB in Pr (Fig. 1b)^37^.

Although, to our knowledge, there is no studies regarding the molecular shapes of plant full-length phytochromes except our own, two SAXS studies have reported the molecular shapes of Bphs. The SAXS model of Bph from *Rhodopseudomonas palustris* displays a Y-shape with two large arms assigned as PSM^50,51^. This arrangement of PSMs resembles the arrangement of the two arms in SAXS models of *P. sativum* LphyA (Fig. 5a,b) and *A. thaliana* phyB (Fig. 1d). In a crystalline state, Bph fragments of *R. palustris* have contact only at OPM pairs, and the PSMs are largely separated from each other^28^. The SAXS model of Bph from cyanobacteria *Synechocystis* has a Y-shape^52^, although the assignment of PSM and OPM is opposite to that of *R. palustris* Bph^50^.

On the other hand, the arrangement of the two subunits in LphyA conflicts with the dimeric association of *A. thaliana* phyB PSM in the crystal structure^12^ as well as with those in the crystal structures of PSM dimers and full-length dimers of various Bphs^25–27,29–35^. To date, three types of association modes of PSMs have been reported in their crystalline state; parallel, anti-parallel, and perpendicular arrangements^30,31,35^ (Fig. 5c). In any of these three modes, parts of a long α-helix harboring the backbone of PSM acts as a major contact site of subunits. The differences between the crystal structures and SAXS models suggest that the association mode of PSM of Bphs and also phyB is sensitive to their primary sequences, composition of functional domains, molecular contacts in crystals, and/or crystallization buffers different from solutions in SAXS. Additionally, it is possible that the association modes of *P. sativum* LphyA and *A. thaliana* phyB dimers in solution are substantially different to those of Bphs.

### Red light induced conformational changes

According to the interpretation of the arrangement of subunits and modules described above (Fig. 5a,b), structural changes of the LphyA molecule occur during the Pr/Pfr interconversion. Then, each subunit shifts from the molecular center by more than 20 Å along the direction of the major axes (Fig. 5d), and the subunit straightens from that in Pr with a bend in the middle by a movement of half of the subunit by more than 20 Å. These conformational changes make *R*g and the maximum dimension of Pfr slightly larger than those of Pr. The positional shift and bend-to-straight change of the subunit exposes molecular surfaces hidden in Pr and reorganizes the surface properties suitable to associate with proteins necessary for nuclear localization and signaling partners, such as PIFs^23^ and ubiquitin ligase^24^.

Since PSMs are separated in LphyA, red light-induced changes with respect to the arrangement of PSM differ from those observed in crystal structures of dimeric PSM of Bphs^25–27,29–35^. However, the arrangement of domains in the PSM subunit may have intrinsic contribution to the change of subunits in the Pr/Pfr conversion of LphyA. In this regard, the normal mode analysis for the crystal structure of a PSM subunit (Fig. 4) provides possible motions in PSM and their relevance to the bending-to-straight change of the subunit shape in Pr-to-Pfr conversion (Fig. 5d). Therefore, PSMs, which have similar architecture among plant and bacterial phytochromes, may commonly possess intrinsic mechanical properties to induce the hinge-bending motion of PHY against the PAS-GAF pair (Fig. 4). The motion would trigger and/or drive OPM relative to PSM and has the potential to straighten the subunit during Pr-to-Pfr conversion (Fig. 5a,b,d). The movement of the PHY in the normal mode analysis may correlate with the PHY movement in PSM of *Deinococcus radiodurans* Bph^29^, and the OPM movement of *Synechocystis* Bph^52^ in their Pr-Pfr phototransformation.

At present, the crystallization of LphyA is difficult, and cryoTEM may be the only technique for visualizing the structure at sub-atomic resolution. In the structure analysis of Pfr, because the specimen solution under red light irradiation is a mixture of Pr and Pfr, molecular images of Pfr must be selected exclusively from noisy TEM images. Then, the present SAXS models of Pr and Pfr would accelerate the selection of Pfr images.

### Structural relevance between LphyA and phyB

From the present and previous^37^ SAXS studies on plant phytochromes, we obtain three low-resolution structure models; a four-leaf shape for *P. sativum* LphyA in Pr, a butterfly shape for *P. sativum* LphyA in Pfr, and a cross shape for *A. thaliana* phyB in Pr. The models are topologically consistent with respect to the shape and arrangement of subunits and modules. The subunits have rod shapes with similar dimensions and are associated at the middle. The angle between the major axes of the subunits depends on the primary sequences and/or Pr/Pfr state. The different association modes of the subunits may influence the different roles of phyA and phyB, contributing to interactions with different proteins.

The SAXS model of phyB is limited to Pr because the SAXS profile from phyB in the steady state displays photo-reversible aggregation^37^. The similar subunit shape, a long rod with a bend in the middle, in the Pr forms of *P. sativum* LphyA and *A. thaliana* phyB suggests that phyB displays red light-induced conformational changes similar to those of LphyA. In vitro, while *P. sativum* phyA in the Pfr-Pfr homodimer is monodispersive (Fig. 2), the Pfr-Pfr homodimer of *A. thaliana* phyB with aggregation properties is suitable for molecular interactions^37^. These differences in the molecular properties of Pfr between phyA and phyB in vitro may correlate with their molecular interactions, the lability/stability of their Pfr, and the lifetimes of Pfr-Pfr homodimer and Pfr-Pr heterodimer in the dark reversion in vivo^53^.

## Materials and methods

### Specimen preparation and SAXS measurements

LphyA was purified under dim green safety light from seven days-old etiolated seedlings of pea (*P. sativum* cv. Alaska) using DEAE-agarose, hydroxyapatite, and immunoaffinity chromatography, as described previously^39^. Purified LphyA was suspended in 25 mM of HEPES and 1 mM of Na_2_EDTA (pH 7.8) and concentrated by ultra-filtration for SAXS measurements. The purity of LphyA was greater than 98%, as determined by Coomassie brilliant blue staining after sodium dodecyl sulfate–polyacrylamide gel electrophoresis and the absorption spectra.

SAXS measurements for a LphyA solution of 1.5–4.0 mg/ml were performed at BL40B2 of SPring-8 using an R-axis IV^++^ (RIGAKU, Japan) system as a detector^39^. The X-ray wavelength and camera distance were 1.000 Å and 1050 mm, respectively. The exposure time was 60 s at 296 K. Each SAXS pattern was normalized by the intensity of the incident X-ray beam monitored by an ionization chamber. From a LphyA solution, SAXS patterns were collected at least in the dark and under red light irradiation as described previously. To ensure a photo-steady state, the specimen solutions were pre-irradiated with red light for 4 min prior to X-ray exposure. SAXS of the buffer solutions for background was recorded before or after the measurements of the LphyA solutions. Little radiation damage, even after 5 min of exposure, was confirmed by UV–visible absorption spectra.

### SAXS data processing and analysis

After the two-dimensional SAXS pattern was reduced to the one-dimensional profile, the background scattering of the buffer solution was subtracted^39^. Profiles in a small-angle region were analyzed using the Guinier analysis^54^. In this analysis, the scattering intensity $I\left( {S,C} \right)$ at a scattering vector length S and concentration $C$ is approximated by the zero-angle scattering intensity $I\left( {S = 0,C} \right)$ and the radius of gyration $R{\text{g}}\left( C \right)$ as

3 $$ I\left( {S,C} \right) = I\left( {S = 0,C} \right){\text{ exp}}\left[ { - \frac{{4\pi^{2} }}{3}R{\text{g}}^{2} \left( C \right) \, S^{2} } \right] ,\quad \, S = \frac{2sin\theta }{\lambda } $$

where *λ* is the X-ray wavelength, and *2θ* is the scattering angle.

When the diluted protein solution is monodispersive, $I\left( {S = 0,C} \right)$ and $R{\text{g}}\left( C \right)^{2}$ linearly depend on the concentration under diluted condition^54^ as

4 $$ \frac{KC}{{I\left( {S = 0,C} \right)}} = \frac{1}{{M_{{\text{W}}} }} + 2A_{2} \cdot C $$

5 $$ R{\text{g}}\left( C \right)^{2} = \, R{\text{g}}\left( {C = 0} \right)^{2} - B_{{{\text{if}}}} \cdot C $$

where $K$ is an experimental constant, $M_{{\text{W}}}$ is the apparent molecular weight of the protein, $A_{2}$ is the second virial coefficient. $B_{{{\text{if}}}}$ reflects the mode of intermolecular interactions and has the same sign with $A_{2}$. The distance-distribution function was calculated using the *GNOM* program^55^.

### Molecular shape prediction

The molecular shapes were restored from a SAXS profile as an assembly of DRs with a radius of 3.8 Å by the *GASBOR* program^36^. The difference between the observed ($I_{\exp } \left( S \right)$) and calculated ($I_{{{\text{model}}}} \left( S \right)$) scattering profiles was monitored using the following equation:

6 $$ \chi^{2} = \frac{1}{N - 1}\sum\limits_{j = 1}^{N} {\left[ {\frac{{I_{\exp } \left( {S_{j} } \right) - \alpha \, I_{{{\text{model}}}} \left( {S_{j} } \right)}}{{\sigma \left( {S_{j} } \right)}}} \right]}^{2} $$

where *N* is the number of data points, $S_{j}$ is the scattering vector length of the *j*th data point, *α* is a scale factor, and $\sigma \left( {S_{j} } \right)$ is the statistical error of the experimental profile.

The number of DRs is a predominant factor in minimizing the discrepancy between the experimental and model profiles^43^. The optimum number of DRs to give the smallest *χ*^2^ was surveyed by varying DRs from 900 to 1200 DRs per subunit with an increment of 25 DRs. For each, the *χ*^2^ value was averaged over 14 independent *GASBOR* calculations. For the determined number of DRs, independent *GASBOR* calculations were conducted on a supercomputing system with 560 Intel Xeon CPU X5690 cores (3.7 GHz per core).

The molecular models were analyzed using the multivariate analysis protocol reported previously^43^. Briefly, after superimposing the models regarding their moment of inertia, the spatial distribution of DRs in each model was expressed as the number density in an array of 6 × 6 × 6 Å^3^ voxels. Then, each model was represented as a point in the multidimensional space spanned by the axes, of which the variable was the number density of DRs in the voxels. By applying PCA, the distribution of molecular models in the multidimensional space was projected onto the plane spanned by the first and second eigenvectors. In our experience as reported^43^, in most cases, the first and second eigenvectors are sufficient to visualize the variations among ab initio models. In the present study, two eigenvectors explained more than 15% of the variance of the Pr and Pfr models. The models were classified into 10 groups by the K-means clustering method. The molecular shape representing each group was calculated by averaging the models after superimposition on a reference structure within each group.

### Normal mode analysis

To understand the inherent molecular motions possible in PSM, normal mode analysis^47^ was applied to an elastic network model^48^ representing the structure of subunit A in the PSM dimer of *A. thaliana* phyB (PDB accession code: 4OUR)^12^. In the elastic network model, the structure of the subunit was expressed as an assembly of Cα atoms connected by springs. The Hookean potential for a Cα atom was calculated from springs connecting atoms located within a given cutoff distance. The computation was performed using the custom-made program *ELASTN*. Through trial calculations, we determined the cutoff distance to maximize the correlation between the thermal factors from the analysis and crystal structure. The force constant of the spring was adjusted to equalize the sum of the theoretical factors with that of the experimental ones.
